# Control of Parenthood

**Published:** 1920-10-23

**Authors:** 


### THE PUBLIC WELL-BEING.

### Control of Parenthood.

### A BOOK OF CONTRADICTIONS.

A book about the control of parenthood, which has just been published under the editorship of the Rev. James Marchant,* is one of the most interesting documents on the subject that have yet appeared. It is less like a book than a summary of evidence and opinions taken before a Commission of Inquiry. For that reason, especially in view of the eminence of the persons who have contributed to the discussion, it has a persuasive quality of open-mindedness which will enable the reader to feel that he is presented with all sides of the question impartially, though how far he will be assisted to derive any practical guidance from the opinions expressed, or supply himself with a safe working hypothesis in this most important matter, is difficult to decide. Diametrically opposite views are expressed with almost equal force and with great candour by the following contributors: Professor J. A. Thompson and Professor Leonard Hill, who deal with the question in its biological aspects; Dean Inge and Mr. Harold Cox, who approach it from an economic standpoint; Dr. Mary Scharlieb, the Rev. Dr. Meyer, and Principal A. E. Garvie, who debate its social and religious elements; and Sir Rider Haggard and Dr. Marie Stopes, who treat imperial and racial considerations in the problem.

#### A State Ideal.

There is also a thoughtful introduction by the Bishop of Birmingham, who sums up his conclusions as to the duty of the State in these terms:—

"We must insure that every mother shall have during and after child-bearing the necessary physical and moral health; that every child shall be cared for so that inefficients shall be almost unknown and true citizens shall be the rule; that undue temptations shall not assail the young and that sin shall not prevail; that the diseases incidental to impurity shall not be allowed to rage unchecked; that men and women shall know the dangers which belong to lust; that we shall all understand how we depend one upon the other; that, whether by emigration or other means, care may be had for those to whom here at home the joys of marriage are denied; and, above all, that the State shall bear in mind that upon the recognition of God in all that concerns marriage and parenthood depends the future well-being of the land we love and of the people upon whom it would seem that at the present time rests the greatest responsibility for setting a world-wide example of the highest and best in life."

This is an ideal which is made less easy of attainment by the opposing views of many of the writers of the essays in the subsequent pages. The contradictions frankly and fearlessly expressed are bound to excite controversy among social reformers and members of the medical profession, as well as among the general public. It could hardly be otherwise when there is a fundamental difference of opinion upon the basic issues (1) whether it is necessary to restrict the population; (2) whether, in any case, it is desirable or right to limit artificially the birth-rate.

#### The Malthusian Theory.

Let us take, by way of a typical contrast, the well-known theory, here very ably put forward, of Mr. Harold Cox and the very conservative outlook of so conscientious and cautious an expert as Dr. Mary Scharlieb. Mr. Cox, as a devout follower of Malthus, plainly asserts that no rate of increase in the population, however low, can be maintained indefinitely, and he urges that those who wish to give an honest answer to an eternal problem ought to be willing to say to-day by what means they think the rate of increase in the English race or of the human race ought to be reduced when the necessity for reduction can no longer be denied. No speculation about the unexhausted possibilities of the globe, he declares, justify the refusal of the anti-Malthusians to answer this question: "Do you wish the potential rate of increase of population to be kept down by a prudential control of the birth-rate or by a punitive expansion of the death-rate?" Finally, he says the real weight of the opposition to birth-control comes from a section of the clergy of the Church of England and from practically all the clergy of the Church of Rome, whose attitude he attacks with characteristic vigour as not being consistent even with Christian morals.

#### A Conservative View.

On the other hand, Dr. Scharlieb is quite as emphatic. Limitation of families is wrong and dangerous, in her opinion, because it does not control nor discipline sexual passion, but by removing the fear of consequenecs it does away with the chief controlling and steadying influence of sexual life. Secondly, "the limitation of the family is not really in the interest of over-burdened mothers." It may relieve them of too frequently recurring child-bearing, and from the burden of too large a household, but on the other hand, it destroys the wife's protection by removing the chief check on the husband's demands. Thirdly, the conditions thus created will tend to the over-development of the sexual side of the character of both man and woman. Dr. Scharlieb then presents a horrible picture of a society fully developed on a recognised basis of birth control. As to the danger of over-population and under-production she asserts, rather vaguely it must be confessed, that the time is not near at hand and that "the resources of science have yet to be devoted to the extraction of the many undeveloped gifts of nature." Intensive cultivation, chemical and electrical, is

* *The Control of Parenthood.* (Putnam's Sons, Bedford Street. 7s. 6d. net.)

THE PUBLIC WELL-BEING—(*continued*).

in its infancy, and any talk of insufficient production is premature. At present we are as an Empire suffering from insufficient population. Her conclusion, therefore, is that the majority of those who advance the arguments in favour of birth control are thinking, not of the right and wrong involved by limitation of families, nor of the spiritual, moral, and nervous injury that might be inflicted by so doing, but that they are chiefly considering the question "from the physical, the hedonistic, and material point of view."

We hope to have an opportunity of referring again to the diverse views of this quite remarkable collection of opinions, but it is sufficient at the moment to point out that what scientific and political and social reformers have to deal with now is not an abstract theory or an academic view of ethics, but with a pressing and difficult problem of human existence that is already seriously affecting the life of the nation from the top to the bottom, a fact which Dr. Scharlieb seems to ignore in her able but unconstructive criticisms. Birth control is being increasingly practised throughout civilisation. The point of immediate concern is how such control shall be wisely directed, so as to avoid its admitted dangers and regulate benevolently our social relationships.

